# Amiloidose Cardíaca por Transtirretina Hereditária Associada a Duas Variantes Patogênicas no Gene TTR

**DOI:** 10.36660/abc.20250651

**Published:** 2026-04-30

**Authors:** Victor Hugo Bonfim, Matheus Martins Monteiro, Moisés Abtibol Machado, Orlando Pereira da Silva, Katia do Nascimento Couceiro, Fábio Fernandes, João Marcos Bemfica Barbosa Ferreira

**Affiliations:** 1 Universidade do Estado do Amazonas Manaus AM Brasil Universidade do Estado do Amazonas, Manaus, AM – Brasil; 2 Hospital das Clínicas da Faculdade de Medicina da Universidade de São Paulo Instituto do Coração São Paulo SP Brasil Instituto do Coração do Hospital das Clínicas da Faculdade de Medicina da Universidade de São Paulo, São Paulo, SP – Brasil

**Keywords:** Amiloidose, Mutação, Genes

## Introdução

A amiloidose engloba um grupo de doenças raras caracterizadas pelo acúmulo^1^ anômalo de proteínas mal dobradas nos tecidos e órgãos. Essas proteínas adotam uma estrutura altamente estável em formato de folhas pregueadas do tipo beta, formando fibrilas insolúveis que se depositam no espaço extracelular. A classificação da amiloidose varia conforme a proteína precursora e o tipo de polímero formado, resultando em diferentes subtipos da doença, cada um com manifestações clínicas distintas.^1^

A amiloidose transtirretina (ATTR) tem dois subtipos primários: ATTR do tipo selvagem (ATTR tipo selvagem, ou ATTRw), anteriormente chamada de "senil" e ATTR variante (ATTR hereditário ou ATTRh), onde os indivíduos possuem variantes patológicas no gene da transtirretina. Até o momento, mais de 120 variantes foram identificadas. Os sintomas clínicos podem se apresentar principalmente como neuropáticos, cardíacos ou uma combinação de ambos.^1^

A mutação Val142Ile é a mais comum da amiloidose hereditária por transtirretina (ATTRh) nos Estados Unidos, sendo identificada em aproximadamente 3 a 4% da população afro-americana. Geralmente, manifesta-se na sétima década de vida, associada a sintomas de insuficiência cardíaca. Já no Brasil e em outras partes do mundo, a mutação mais prevalente é a Val50Met, frequentemente relacionada a manifestações neurológicas e sistêmicas da doença. Já a variante p.Asp58Ala da TTR (também referida como Asp38Ala) é rara globalmente, há relatos de predominância em coortes coreanas, mas permanece incomum em outras populações.^2,3^ Na tabela 1, resumimos as principais características clínicas das variantes presentes na paciente descrita.

**Tabela 1:** Características clínicas das variantes Asp58Ala e Val142Ile

	Asp58Ala.	Val142Ile
Idade	Por volta de 60 anos	Por volta de 70 anos
Prevalência	55% em pacientes da Coreia do Sul (rara em outros continentes)^5^	3 a 4% de afrodescendentes nos Estados Unidos da América^4^
Fenótipo prevalente	Misto	Cardiopatia
Manifestações neurológicas	Frequentes	Raras
Manifestações autonômicas	Frequentes	Raras
Manifestações cardíacas	Frequentes	Frequentes
Manifestações gastrointestinais	Frequentes	Raras

Fonte: elaborada pelo autor, Bonfim, 2025.

Relatamos as manifestações clínicas e a progressão da amiloidose TTR em uma paciente do sexo feminino com duas variantes patogênicas do gene TTR, Asp58Ala e Val142Ile. Esta paciente faz parte de um projeto maior, aprovado pelo CEP da Universidade do Estado do Amazonas com o número do CAAE:55200322.5.3009.516.

## Relato de Caso

Paciente, do sexo feminino, 60 anos, afrodescendente, proveniente do interior do estado do Amazonas, procurou atendimento médico apresentando sintomas como parestesias de membros superiores e inferiores, vertigem, precordialgia atípica, hipotensão arterial e perda ponderal, com início cerca de um ano antes do atendimento inicial. Informou ter sofrido ao menos cinco episódios de síncope, sem pródromos e com estabelecimento total da consciência logo após o episódio. Apresentou também diarreia intermitente associada a uma significativa perda de peso de 20 kg no último ano. No momento da consulta, sua pressão arterial foi aferida em 51x36 mmHg. A ausculta cardíaca e respiratória se encontrava normal. Foi atendida em consultório de neurologia e cardiologia, tendo sido feito diagnóstico clínico inicial de neuropatia e cardiopatia a esclarecer.

**Figura 1:**
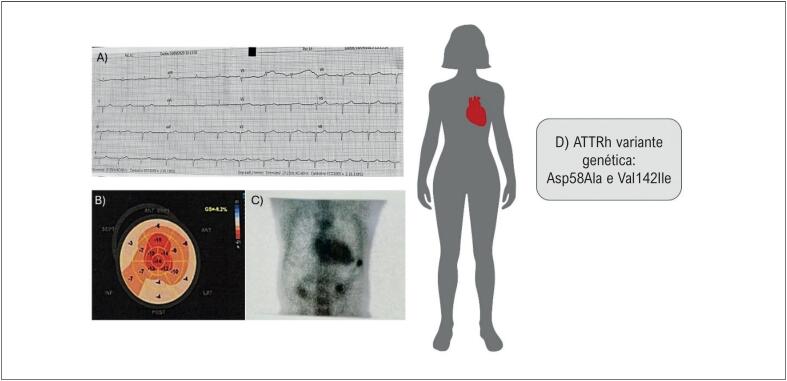
Amiloidose cardíaca em paciente do sexo feminino com duas variantes genéticas do gene transtirretina (TTR). A) Eletrocardiograma demonstrando baixa voltagem QRS (Plano Frontal); B) Eco com strain demonstrando apical sparring; C) Presença de captação do pirofosfato em área cardíaca. Escala de Perugini = grau 3; D) Amiloidose por transtirretina hereditária: Variantes genéticas identificadas Asp58Ala e Val142Ile.

Durante a investigação ambulatorial, o eletrocardiograma: Ritmo sinusal, condução atrioventricular normal, FC=79 bpm, desvio do eixo para a esquerda, demonstrou baixa voltagem de complexos QRS no plano frontal e ausência de progressão de onda R na parede anterior, achado característico de amiloidose cardíaca (Figura 1A). Realizou Holter-24 horas sem alterações significativas. Raio-X com área cardíaca normal e presença de congestão pulmonar

O ecocardiograma demostrou o diâmetro diastólico do VE de 37 mm, fração de ejeção do VE de 54%, volume atrial indexado 60 ml/m^2^, aumento da espessura das paredes do ventrículo esquerdo com parede posterior do ventrículo esquerdo 17 mm, septo 16 mm, massa ventricular indexada de 163 g/ m^2^, espessamento das valvas mitras e aórticas e strain global longitudinal reduzido (-9,2%) com preservação da deformidade nos segmentos apicais (Figura 1B), alterações compatíveis com quando de amiloidose cardíaca. Apresentava disfunção diastólica grau III (tipo restritivo) (Figura 1C). Não realizou ressonância magnética cardíaca e/ou biópsia endomiocárdica devido à falta de acesso pelo sistema público no Amazonas.

Estes exames levaram a suspeita de amiloidose cardíaca. Foi descartada a forma AL após a realização de exames específicos, incluindo imunofixação sérica e urinária, dosagem da relação Kappa/Lambda. A cintilografia cardíaca com pirofosfato evidenciou captação grau III, altamente sugestiva de amiloidose cardíaca do tipo transtirretina (figura 1C).

A análise genética por sequenciamento do gene TTR identificou duas variantes distintas: a variante chr18:31.592.999, responsável pela substituição do asparto no códon 58 por alanina, e a variante chr18:31.598.655, que provoca a troca de valina por isoleucina na posição 142 (Figura 1D).

Foi iniciado tratamento com fludrocortisona 0,1 mg ao dia, gabapentina 300 mg de 8/8 horas, dapagliflozina 10 mg ao dia e Tafamidis meglumina de 20 mg/dia que foi a dose disponibilizada pelo Sistema Único de Saúde. A paciente não teve acesso à dose de 80 mg preconizada para a forma cardíaca durante o acompanhamento. A paciente evoluiu para óbito por morte súbita, cerca de 4 meses após o início do tratamento. Diante do caráter hereditário da condição, foi recomendado o aconselhamento genético para os familiares, visando ao rastreamento precoce e manejo adequado da doença.

## Discussão

A variante Asp58Ala (ou p.Asp38Ala, D38A) é considerada rara entre as variantes encontradas de amiloidose hereditária na maioria dos continentes. No entanto, é mais encontrada em pacientes asiáticos, sendo a mais frequente em estudo multicêntrico realizado na Coreia do Sul.^4^ Está associada a sintomas como hipotensão ortostática, diarreia crônica e polineuropatia periférica, podendo apresentar também acometimento cardíaco.^5^ Até o momento, não há mortalidade estratificada para a variante TTR p.Asp38Ala/p.Asp58Ala As séries coreanas identificam sua predominância, mas sem dados de sobrevida por genótipo.

A variante Val142Ile (também conhecida como p.Val122Ile ou V122I), predominante em indivíduos de ascendência africana, é a principal causa de ATTR hereditária, manifestando-se principalmente como cardiomiopatia na sétima ou oitava década de vida. Está associada a maiores taxas de insuficiência cardíaca e a neuropatia autonômica é rara ou ausente.^6^

A paciente apresentou sintomas compatíveis com disfunção autonômica, tais como diarreia e hipotensão arterial, quadro frequentemente observado em indivíduos com ATTRh de início precoce, enquanto nos casos de manifestação tardia, essa alteração tende a ser menos evidente. Além disso, relatou perda de peso involuntária, um sintoma que pode surgir nos estágios iniciais da doença. O comprometimento cardíaco está presente na maioria dos casos e pode se manifestar por aumento da espessura das paredes ventriculares, distúrbios na condução elétrica ou arritmias diversas. Em pacientes com ATTRh de manifestação tardia, a cardiomiopatia costuma ser um achado precoce, sendo a principal característica da variante Val142Ile.^3^

De acordo com o ensaio ATTR-AC, em pacientes com amiloidose cardíaca, a dose de 80 mg do Tafamidis associado ao sal meglumina demonstrou maior sobrevida em comparação com a dose de 20 mg (esta utilizada na forma neurológica). Existe também a dose de 61 mg do Tafamidis livre que possui bioequivalência com a dose de 80 mg do Tafamidis com meglumina. No caso relatado a paciente obteve apenas acesso a dose de Tafamidis com meglumina de 20 mg/dia, de acordo com as políticas públicas do Sistema Único de Saúde. Além disso, as terapias de silenciamento da TTR, RNAi (patisiran) e antissenso (inotersen) mostraram, em fase 3 para ATTRv com polineuropatia, redução da progressão neurológica e sinais favoráveis na cardiomiopatia.^7^

No presente relato, pode ser sugerido que a manifestação cardíaca da doença pode ter sofrido predomínio de evolução da variante Val142Ile, com provável interferência da variante Asp58Ala, ocasionando um acometimento cardíaco grave mais precoce. As manifestações neurológicas e autossômicas parecem ter ocorrido por interferência da variante Asp58Ala.

A manifestação clínica da doença pode ser significativamente influenciada pela existência de duas variantes. Pesquisas indicaram a presença de mutações protetoras que podem atrasar ou diminuir os sintomas. Matsumoto et al. relataram um caso de amiloidose de início tardio envolvendo um composto heterozigoto Val30Met/Lys80Arg.^8^ Terazaki et al. documentaram um caso envolvendo um portador de Val30Met (p.Val50Met) e Arg104His que exibiu sintomas mínimos da doença.^9^ Por outro lado, foi relatado um caso de associação das variantes p.Val30Met e p.Val122Ile no gene TTR, no qual o paciente apresentou comprometimento neuropático, cardíaco e renal. A concomitância de duas mutações patogênicas pode ter contribuído para a manifestação clínica mais grave e a progressão acelerada da doença.^10^

## Conclusão

Apesar de ambas as variantes estarem associadas à ATTRh de forma isolada, não se encontrou registro na literatura da ocorrência simultânea das variantes p.Asp58Ala e p.Val142Ile em um mesmo indivíduo. Cada variante apresenta um perfil clínico específico, sendo que a p.Asp58Ala está relacionada a um acometimento sistêmico mais amplo, enquanto a p.Val142Ile se manifesta predominantemente com alterações cardíacas. No presente relato, acredita-se que o acometimento cardíaco pode ter ocorrido em decorrência da variante Val142Ile com interferência da variante Asp58Ala levando a um acometimento mais precoce. As manifestações neurológicas e autossômicas parecem ter ocorrido por interferência da variante Asp58Ala.

## Data Availability

Os conteúdos subjacentes ao texto da pesquisa estão contidos no manuscrito.
