# Supplementary material for: Amiloidose Cardíaca por Transtirretina Hereditária Associada a Duas Variantes Patogênicas no Gene TTR
Source: Arq Bras Cardiol. 2026 Apr 30;123(4):e20250651. [Article in Portuguese] doi: 10.36660/abc.20250651 (PMC13155777; doi:10.36660/abc.20250651)
Supplement: Translation

# Translation: Hereditary Transthyretin Cardiac Amyloidosis Associated with Two Pathogenic Variants in the TTR Gene

**DOI:** 10.36660/abc.20250651i

**Authors:** Victor Hugo Bonfim, Matheus Martins Monteiro, Moisés Abtibol Machado, Orlando Pereira da Silva, Katia do Nascimento Couceiro, Fábio Fernandes, João Marcos Bemfica Barbosa Ferreira

**Affiliations:** 1 Manaus AM Brasil Universidade do Estado do Amazonas, Manaus, AM – Brasil; 2 São Paulo SP Brazil Instituto do Coração do Hospital das Clínicas da Faculdade de Medicina da Universidade de São Paulo, São Paulo, SP – Brazil

**Keywords:** Amyloidosis, Mutation, Genes

## Introduction

Amyloidosis encompasses a group of rare diseases characterized by the accumulation of amyloidosis.^1^ Amyloidosis is an abnormal formation of misfolded proteins in tissues and organs. These proteins adopt a highly stable beta-sheet structure, forming insoluble fibrils that are deposited in the extracellular space. Amyloidosis is classified according to the precursor protein and the type of polymer formed, resulting in different subtypes of the disease, each with distinct clinical manifestations.^1^

Transthyretin amyloidosis (ATTR) has two primary subtypes: wild-type ATTR (wild-type ATTR, or ATTRw), formerly called "senile," and variant ATTR (hereditary ATTR, or ATTRh), where individuals possess pathological variants in the transthyretin gene. To date, more than 120 variants have been identified. Clinical symptoms may present primarily as neuropathic, cardiac, or a combination of both.^1^

The Val142Ile mutation is the most common in hereditary transthyretin amyloidosis (ATTRh) in the United States, being identified in approximately 3 to 4% of the African American population. It generally manifests in the seventh decade of life, associated with symptoms of heart failure. In Brazil and other parts of the world, the most prevalent mutation is Val50Met, frequently related to neurological and systemic manifestations of the disease.AlreadyThe p.Asp58Ala variant of TTR (also referred to as Asp38Ala) is rare globally; there are reports of prevalence in Korean cohorts, but it remains uncommon in other populations.^2.3^Table 1 summarizes the main clinical characteristics of the variants present in the patient described.

**Table 1:** Clinical characteristics of the Asp58Ala and Val142Ile variants

	Asp58Ala.	Val142Ile
Age	Around 60 years old	Around 70 years old
Prevalence	55% in patients from South Korea (rare in other continents)^5^	3 to 4% of African Americans in the United States of America^4^
Prevalent phenotype	Mixed	Heart disease
Neurological manifestations	Frequent	Rare
Autonomous manifestations	Frequent	Rare
Cardiac manifestations	Frequent	Frequent
Gastrointestinal manifestations	Frequent	Rare

Source: compiled by the author, Bonfim, 2025.

We report the clinical manifestations and progression of TTR amyloidosis in a female patient with two pathogenic variants of the TTR gene, Asp58Ala and Val142Ile. This patient is part of a larger project, approved by the Ethics Committee of the State University of Amazonas under CAAE number: 55200322.5.3009.516.

## Case Report

A 60-year-old female patient of African descent, from the interior of the state of Amazonas, sought medical attention, presenting symptoms such as paresthesia of the upper and lower limbs, vertigo, atypical precordialgia, hypotension, and weight loss, which began approximately one year before the initial consultation. She reported having suffered at least five episodes of syncope, without prodromal symptoms and with full restoration of consciousness immediately after the episode. She also presented intermittent diarrhea associated with a significant weight loss of 20 kg in the last year. At the time of the consultation, her blood pressure was measured at 51x36 mmHg. Cardiac and respiratory auscultation were normal. She was seen in a neurology and cardiology clinic, where an initial clinical diagnosis of neuropathy and cardiomyopathy was made, which was to be clarified.

During the outpatient investigation, the electrocardiogram showed: Sinus rhythm, normal atrioventricular conduction, HR=79 bpm, left axis deviation, demonstrated low voltage QRS complexes in the frontal plane, and absence of R wave progression in the anterior wall, a characteristic finding of cardiac amyloidosis (Figure 1A). A 24-hour Holter monitor showed no significant changes. Chest X-ray showed a normal cardiac area and pulmonary congestion.

**Figure 1:** Cardiac amyloidosis in a female patient with two genetic variants of the transthyretin (TTR) gene. A) Electrocardiogram showing low QRS voltage (Frontal Plane); B) Echocardiogram with strain showing apical sparring; C) Presence of pyrophosphate uptake in the cardiac area. Perugini scale = grade 3; D) Hereditary transthyretin amyloidosis: Genetic variants identified Asp58Ala and Val142Ile.

The echocardiogram showed a left ventricular diastolic diameter of 37 mm, left ventricular ejection fraction of 54%, indexed atrial volume of 60 ml/m^2^, increased left ventricular wall thickness with a left ventricular posterior wall thickness of 17 mm, septum thickness of 16 mm, indexed ventricular mass of 163 g/m^2^, thickening of the mitral and aortic valves, and reduced global longitudinal strain (-9.2%) with preservation of the deformity in the apical segments (Figure 1B), changes compatible with cardiac amyloidosis. The patient presented with grade III diastolic dysfunction (restrictive type) (Figure 1C). Cardiac magnetic resonance imaging and/or endomyocardial biopsy were not performed due to a lack of access through the public healthcare system in Amazonas.

These tests led to suspicion of cardiac amyloidosis. The AL form was ruled out after specific tests, including serum and urine immunofixation, and measurement of the Kappa/Lambda ratio. Cardiac scintigraphy with pyrophosphate showed grade III uptake, highly suggestive of transthyretin-type cardiac amyloidosis (Figure 1C).

Genetic analysis by sequencing of the TTR (transthyretin) gene identified two distinct variants: the chr18:31.592.999 variant, responsible for the substitution of aspart at codon 58 for alanine, and the chr18:31.598.655 variant, which causes the exchange of valine for isoleucine at position 142 (Figure 1D).

Treatment was initiated with fludrocortisone 0.1 mg daily, gabapentin 300 mg every 8 hours, dapagliflozin 10 mgdaily, and tafamidis meglumine 20 mg daily, which was the dose provided by the Brazilian Unified Health System (SUS). The patient did not have access to the 80 mg dose recommended for the cardiac form during follow-up. The patient died suddenly approximately 4 months after the start of treatment. Given the hereditary nature of the condition, genetic counseling was recommended for family members to allow for early screening and appropriate management of the disease.

## Discussion

The Asp58Ala variant (or p.Asp38Ala, D38A) is considered rare among the variants of hereditary amyloidosis found on most continents. However, it is more commonly found in Asian patients, being the most frequent in a multicenter study conducted in South Korea.^4^ It is associated with symptoms such as orthostatic hypotension, chronic diarrhea, and peripheral polyneuropathy, and may also present with cardiac involvement.^5^ Currently, there is no stratified mortality data for the TTR p.Asp38Ala/p.Asp58Ala variant. Korean series identify their predominance, but without survival data by genotype.

The Val142Ile variant (also known as p.Val122Ile or V122I), predominant in individuals of African descent, is the main cause of hereditary transthyretin amyloidosis, manifesting primarily as cardiomyopathy in the seventh or eighth decade of life. It is associated with higher rates of heart failure, and autonomic neuropathy is rare or absent.^6^

The patient presented symptoms consistent with autonomic dysfunction, such as diarrhea and hypotension, a condition frequently observed in individuals with early-onset hATTRh, while in late-onset cases, this alteration tends to be less evident. In addition, she reported unintentional weight loss, a symptom that can appear in the early stages of the disease. Cardiac involvement is present in most cases and can manifest as increased ventricular wall thickness, electrical conduction disturbances, or various arrhythmias. In patients with late-onset hATTRh, cardiomyopathy is usually an early finding, being the main characteristic of the Val142Ile variant.^3^

According to the ATTR-AC trial, in patients with cardiac amyloidosis, the 80 mg dose of Tafamidis combined with meglumine salt demonstrated greater survival compared to the 20 mg dose (used in the neurological form). There is also a 61 mg dose of free Tafamidis that has bioequivalence with the 80 mg dose of Tafamidis with meglumine. In the reported case, the patient only had access to the 20 mg/day dose of Tafamidis with meglumine, according to the public policies of the Unified Health System (SUS). Furthermore, TTR silencing therapies, RNAi (patisiran) and antisense (inotersen), showed, in phase 3 for vATTR with polyneuropathy, a reduction in neurological progression and favorable signs in cardiomyopathy.^7^

In this report, it can be suggested that the cardiac manifestation of the disease may have been predominantly caused by the Val142Ile variant, with probable interference from the Asp58Ala variant, resulting in earlier, more severe cardiac involvement. The neurological and autosomal manifestations appear to have occurred due to interference from the Asp58Ala variant.

The clinical manifestation of the disease can be significantly influenced by the presence of two variants. Research has indicated the presence of protective mutations that may delay or lessen the symptoms. Matsumoto et al. reported a case of late-onset amyloidosis involving a heterozygous Val30Met/Lys80Arg compound.^8^ Terazaki et al. documented a case involving a carrier of Val30Met (p.Val50Met) and Arg104His who exhibited minimal symptoms of the disease.^9^ On the other hand, a case of association between the p.Val30Met and p.Val122Ile variants in the TTR gene was reported, in which the patient presented with neuropathic, cardiac, and renal impairment. The concomitance of two pathogenic mutations may have contributed to the more severe clinical manifestation and accelerated disease progression.^10^

## Conclusion

Although both variants are associated with ATTRh in isolation, no record has been found in the literature of the simultaneous occurrence of the p.Asp58Ala and p.Val142Ile variants in the same individual. Each variant presents a specific clinical profile, with p.Asp58Ala being related to a broader systemic involvement, while p.Val142Ile manifests predominantly with cardiac alterations. In the present report, it is believed that cardiac involvement may have occurred as a result of the Val142Ile variant with interference from the Asp58Ala variant, leading to earlier onset. The neurological and autosomal manifestations appear to have occurred due to interference from the Asp58Ala variant.

## Data Availability

The underlying content of the research text is contained within the manuscript.
